# PCSK9 Enhances Cardiac Fibrogenesis via the Activation of Toll-like Receptor and NLRP3 Inflammasome Signaling

**DOI:** 10.3390/ijms26051921

**Published:** 2025-02-23

**Authors:** Cheng-Chih Chung, Yu-Hsun Kao, Yao-Chang Chen, Yung-Kuo Lin, Satoshi Higa, Kai-Cheng Hsu, Yi-Jen Chen

**Affiliations:** 1 Division of Cardiology, Department of Internal Medicine, School of Medicine, College of Medicine, Taipei Medical University, Taipei 110, Taiwan; michaelchung110@gmail.com (C.-C.C.); yklin213@yahoo.com.tw (Y.-K.L.); 2 Division of Cardiovascular Medicine, Department of Internal Medicine, Wan Fang Hospital, Taipei Medical University, Taipei 116, Taiwan; 3 Taipei Heart Institute, Taipei Medical University, Taipei 110, Taiwan; 4 Graduate Institute of Clinical Medicine, College of Medicine, Taipei Medical University, Taipei 11031, Taiwan; yuhsunkao@gmail.com; 5 Department of Medical Education and Research, Wan Fang Hospital, Taipei Medical University, Taipei 11031, Taiwan; 6 Department of Biomedical Engineering, National Defense Medical Center, Taipei 11490, Taiwan; yaochang.chen@gmail.com; 7 Cardiac Electrophysiology and Pacing Laboratory, Division of Cardiovascular Medicine, Makiminato Central Hospital, Okinawa 1199, Japan; sa_higa@yahoo.co.jp; 8 Graduate Institute of Cancer Biology and Drug Discovery, College of Medical Science and Technology, Taipei Medical University, Taipei 110, Taiwan; 9 Ph.D. Program for Cancer Molecular Biology and Drug Discovery, College of Medical Science and Technology, Taipei Medical University, Taipei 110, Taiwan

**Keywords:** fibroblasts, fibrosis, PCSK9, NLRP3, toll-like receptor, inflammasome

## Abstract

Proprotein convertase subtilisin/kexin type 9 (PCSK9) has emerged as a novel target for reducing low-density lipoprotein cholesterol. PCSK9 activates the atherosclerosis process through pro-inflammation signaling. Furthermore, the serum level of PCSK9 is positively correlated with mortality in patients with heart failure (HF). Cardiac fibrosis plays a crucial role in the pathophysiology of HF. In this study, we intended to examine whether PCSK9 can increase fibroblast activities and explore what its underlying mechanisms are. Migration, proliferation analyses, and Western blotting were used on human cardiac fibroblasts with and without PCSK9. Alirocumab (a PCSK9 inhibitor, 10 mg/kg/week intra-peritoneally for 28 consecutive days) was treated in isoproterenol (100 mg/kg, subcutaneous injection)-induced HF rats. PCSK9 (50, 100 ng/mL) increased proliferation, myofibroblast differentiation capability, and collagen type I production. Compared with control cells, PCSK9 (100 ng/mL)-treated cardiac fibroblasts showed higher nucleotide-binding domain (NOD)-like receptor protein 3 (NLRP3), interleukin (IL)-1, myofibroblast differentiation, and collagen production capabilities, which were attenuated by MCC950 (an NLRP3 inhibitor, 100 μmol/L). PCSK9 upregulated Myd88 and NF-κB signaling, which were reduced by TAK242 (a toll-like receptor (TLR) 4 inhibitor, 10 μmol/L). Moreover, alirocumab significantly improved left ventricular systolic function and attenuated fibrosis in HF rats. In conclusion, PCSK9 upregulates NLRP3 signaling and the profibrotic activities of cardiac fibroblasts through the activation of TLR4/Myd88/NF-κB signaling.

## 1. Introduction

Proprotein convertase subtilisin/kexin type 9 (PCSK9) has emerged as a novel target for reducing low-density lipoprotein (LDL) cholesterol. A gain-of-function mutation in the PCSK9 gene has been recognized in patients with familial hypercholesterolemia, which is correlated with the increased incidence and mortality rate of myocardial infarction (MI) [1,2,3]. In contrast, patients with a loss-of-function mutation in PCSK9 genes have less cardiovascular event risk [4]. The plasma level of PCSK9 is elevated in patients post-MI [5] and can predict the worsening of left ventricular (LV) systolic function 6 months later [6]. PCSK9 inhibitor improved LV systolic function of post-MI mice by reducing cardiac autophagy [7]. Alirocumab, a novel PCSK9 monoclonal antibody, can decrease cardiac events and mortality in patients with MI [8]. A higher plasma PCSK9 level is associated with an increased risk of all-cause mortality in patients with heart failure (HF) [9]. Adverse LV remodeling is regarded as an intermediate phenotype in the progression of HF [10]. In patients with HF, increased cardiac fibrosis is linked to poorer outcomes [11]. A previous study revealed that the fibrotic inhomogeneity of LV tissue is highly correlated with the cardiac event post-MI [12]. Patients with the PCSK9 loss-of-function variant have shown higher protection against liver fibrosis. However, whether and how PCSK9 modulates cardiac fibrogenesis remains unclear.

Higher inflammatory markers are significantly increased in HF patients and are positively correlated with the severity of the disease [13]. Since inflammation signaling pathways play a crucial role in cardiac fibrosis [14], HF may induce various damage-associated molecular patterns (DAMPs) and activate nucleotide-binding domain, leucine-rich-containing family, pyrin domain-containing-3 (NLRP3) inflammasome through pattern recognition receptors such as toll-like receptor (TLR), thereby triggering cardiac fibrosis [15,16,17]. PCSK9 knockdown decreases macrophage accumulation in atherosclerotic plaques and vascular inflammation markers of interleukin (IL)-1β, downstream signaling of NLRP3 [18]. Hypoxic stress, a key contributor to post-MI HF, stimulates the secretion of PCSK9 from cardiomyocytes, which in turn promotes IL-1β secretion from co-cultured macrophages and induces cardiomyocyte apoptosis [19]. Accordingly, PCSK9 may directly activate fibrogenesis by enhancing NLRP3 inflammasome signaling, leading to pro-fibrosis potential. In this study, we intended to examine whether PCSK9 can increase cardiac fibrogenesis and explore what are its underlying mechanisms. We also investigated the effects of PCSK9 inhibition on heart function in HF animals.

## 2. Results

### 2.1. Effects of PCSK9 on the Profibrotic Cellular Activities of Cardiac Fibroblasts

**Figure 1:**
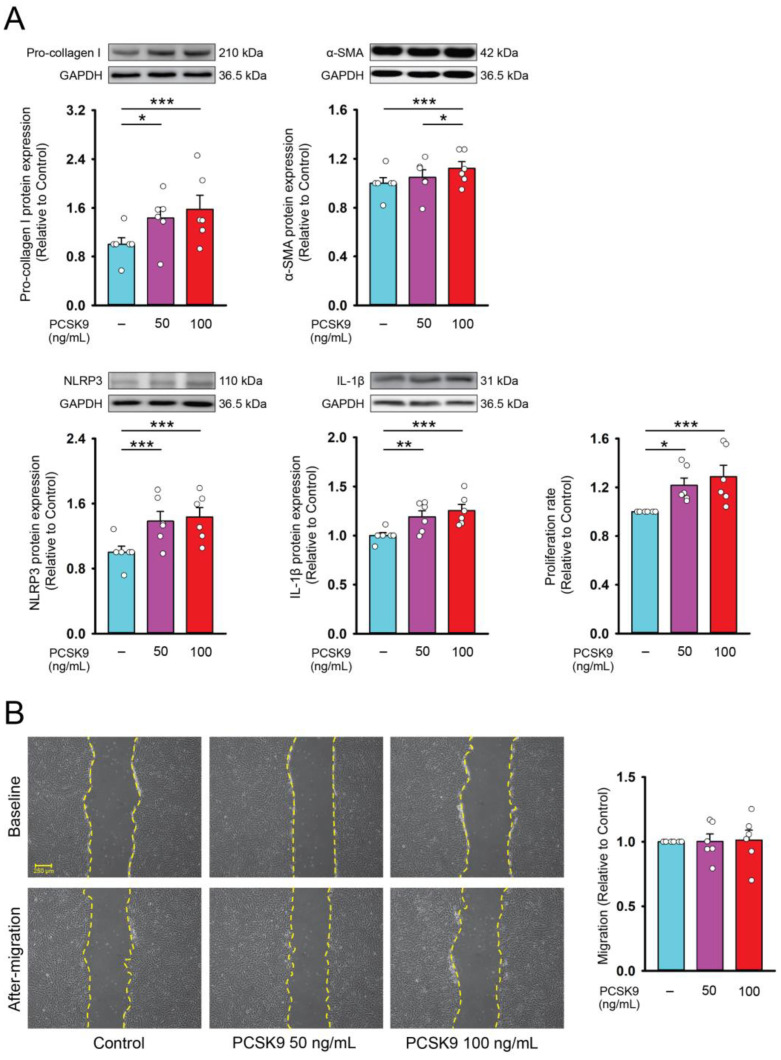
Collagen production, myofibroblast differentiation, cell proliferation, and migration capabilities of cardiac fibroblasts treated with proprotein convertase subtilisin/kexin type 9 (PCSK9). (**A**) Photographs, individual data points, and averaged data revealed expression of pro-collagen type I, and α-smooth muscle actin (SMA), NLR Family Pyrin Domain-Containing 3 (NLRP3), and interleukin (IL)-1β (*n* = 6 independent experiments) and proliferation rate (*n* = 6 independent experiments) of control and PCSK9 (50 or 100 ng/mL)-treated cardiac fibroblasts. GAPDH was used as a loading control. (**B**) Photographs, individual data points, and averaged data revealed the migration assay results of cardiac fibroblasts treated with PCSK9 (50 or 100 ng/mL). The left upper panels display different group’s initial scratch (baseline). Left lower panels displayed the images 6 h after the scratch was created (after migration) (*n* = 6 independent experiments). * *p* < 0.05, ** *p* < 0.01, *** *p* < 0.005.

PCSK9 (50 or 100 ng/mL)-treated fibroblasts had higher pro-collagen type I, α-smooth muscle actin (α-SMA, a myofibroblast differentiation marker), NLRP3, and IL-1β protein expression and proliferation rates as compared with control cells (Figure 1). However, control and PCSK9-treated cardiac fibroblasts exhibited similar migration capabilities.

### 2.2. Inflammation Signaling Pathway in PCSK9-Treated Cardiac Fibroblasts

**Figure 2:**
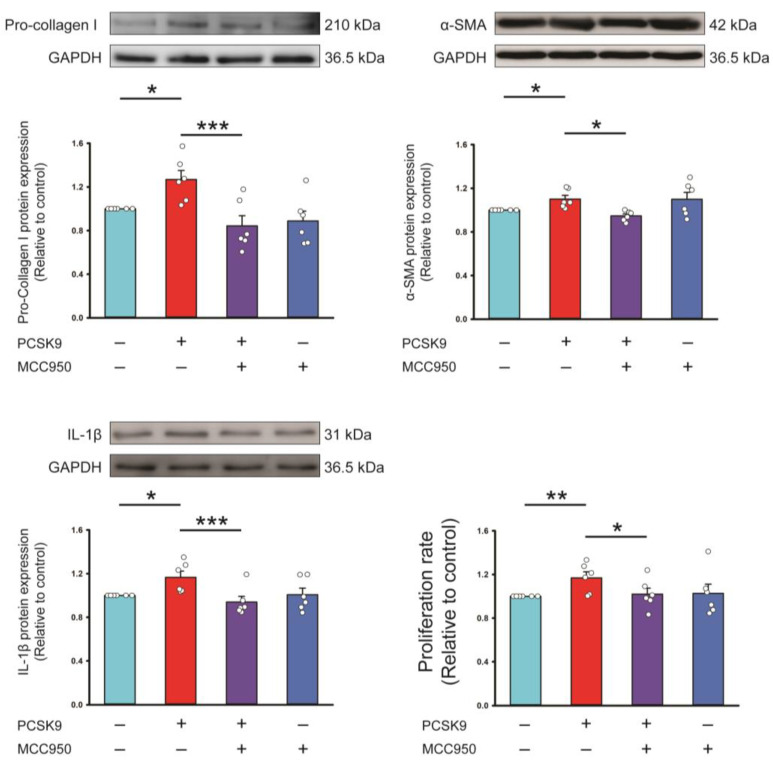
Effects of proprotein convertase subtilisin/kexin type 9 (PCSK9) on NLR Family Pyrin Domain-Containing 3 (NLRP3) and downstream signaling. Photographs, individual data points, and averaged data of the expression of pro-collagen type I, α-smooth muscle actin (SMA), and interleukin (IL)-1β (*n* = 6 independent experiments) and proliferation rate (*n* = 6 independent experiments) of control cells and PCSK9 (100 ng/mL)-treated cardiac fibroblasts cotreated with or without NLRP3 inhibitor (MCC950, 100 μmol/L). GAPDH was used as the loading control. * *p* < 0.05, ** *p* < 0.01, *** *p* < 0.005.

The PCSK9 (100 ng/mL) upregulated protein expressions of procollagen type I, α-SMA, IL-1β protein expression, and proliferation capability, which were attenuated by MCC950 (an NLRP3 inhibitor, 100 μmol/L) (Figure 2).

### 2.3. The Interaction Between PCSK9 with TLR4

**Figure 3:**
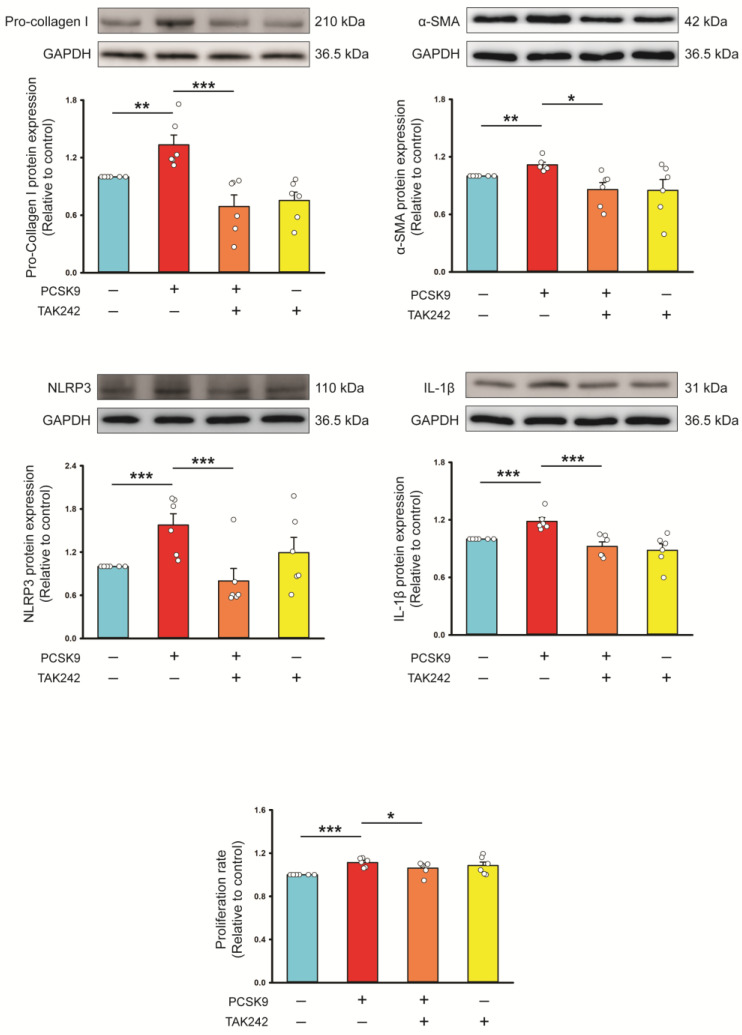
Pro-fibrotic and inflammatory effects of proprotein convertase subtilisin/kexin type 9 (PCSK9) on Toll-like receptor 4 (TLR4) signaling. Photographs, individual data points, and averaged data of the protein expression of pro-collagen type I, α-smooth muscle actin (SMA), NLR Family Pyrin Domain-Containing 3 (NLRP3), and interleukin (IL)-1β (*n* = 6 independent experiments) and proliferation rate (*n* = 6 independent experiments) of control cells and PCSK9 (100 ng/mL)-treated cardiac fibroblasts cotreated with or without TAK-242 (a TLR4 inhibitor, 10 μmol/L). GAPDH was used as the loading control. * *p* < 0.05, ** *p* < 0.01, *** *p* < 0.005.

**Figure 4:**
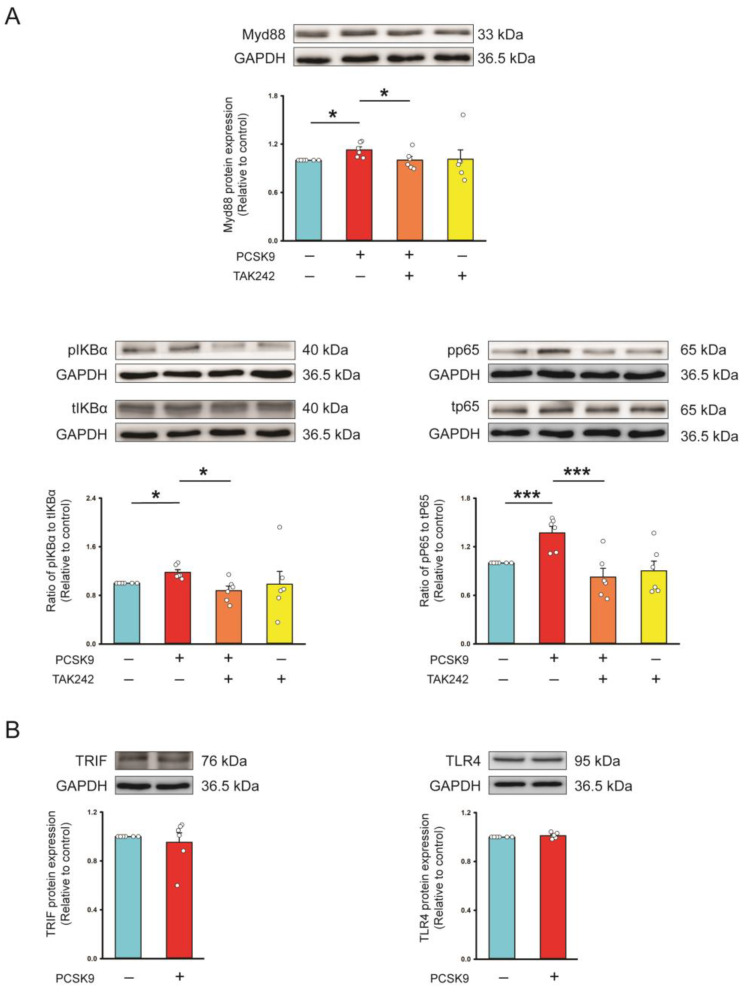
Effects of proprotein convertase subtilisin/kexin type 9 (PCSK9) on Toll-like receptor 4 (TLR4) downstream signaling. (**A**) Photographs, individual data points, and averaged data of the protein expression of Myd88, ratio of phosphorylated (p) to total (t) p65 and IκBα in control cells and PCSK9 (100 ng/mL)-treated cardiac fibroblasts cotreated with or without TAK-242 (a TLR4 inhibitor, 10 μmol/L) (*n* = 6 independent experiments). (**B**) Photographs, individual data points, and averaged data revealed similar TLR4 and Toll-like receptor adapter molecule 1 (TRIF) protein expression in control cells and PCSK9 (100 ng/mL)-treated cardiac fibroblasts (*n* = 6 independent experiments). GAPDH was used as the loading control. * *p* < 0.05, *** *p* < 0.005.

In the presence of TAK-242 (a TLR4 inhibitor, 10 μmol/L), the PCSK9-upregulated pro-collagen type I, α-SMA, NLRP3, IL-1β, Myd88, ratio of phosphorylated p65 and IκBα protein expression and proliferation capability were suppressed, suggesting that PCSK9 can activate profibrotic cellular activities and NLRP3 signaling through TLR4 signaling (Figure 3 and Figure 4). However, control and PCSK9-treated fibroblasts revealed similar TIR-domain-containing adapter-inducing interferon (TRIF) and TLR4 protein expression (Figure 4).

### 2.4. Effects of PCSK9 Inhibitor on Heart Structure, Systolic Function, and Cardiac Fibrosis

**Figure 5:**
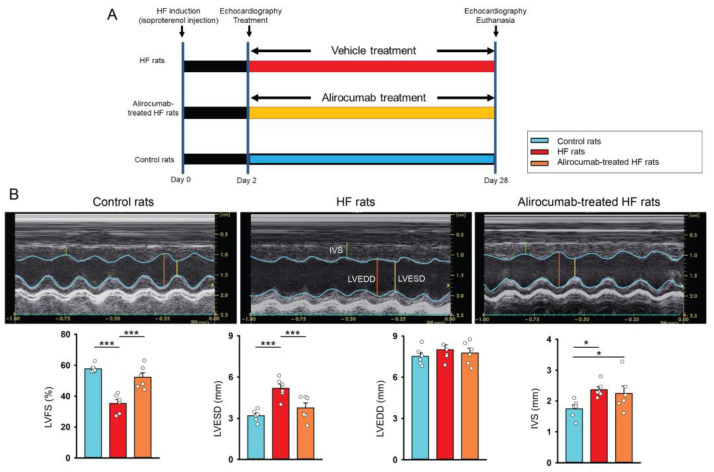
Treatment protocol of proprotein convertase subtilisin/kexin type 9 (PCSK9) inhibitor (alirocumab) and effects of alirocumab on heart structure and systolic function of rats with isoproterenol-induced heart failure (HF). (**A**) Schematic summarizing the treatment protocol for Wistar rats with isoproterenol (100 mg/kg, subcutaneous injection)-induced HF rats, alirocumab (10 mg/kg/week subcutaneously injection for 28 consecutive days)-treated HF rats, and control rats. (**B**) Photographs, individual data points, and averaged data present the results of left ventricular fractional shortening (LVFS), LV end-systolic diameter (LVESD), LV end-diastolic diameter (LVEDD), and interventricular septum (IVS) in control rats (*n* = 6 rats), HF rats (*n* = 6 rats), and alirocumab-treated HF rats (*n* = 6 rats). * *p* < 0.05, *** *p* < 0.005.

Compared to the serum levels of PCSK9 prior to HF induction by isoproterenol, the levels of PCSK9 were significantly elevated 14 days after HF induction (*n* = 6 rats, 178.3 ± 20.8 versus 272.2 ± 20.5 ng/mL, *p* < 0.01). As shown in Figure 5A, we studied the effects of alirocumab (10 mg/kg, a PCSK9 monoclonal antibody) on cardiac structure, systolic function, serum lipid profile, and cardiac fibrosis in vivo. Echocardiography findings revealed that isoproterenol-induced HF rats exhibited lower LV systolic function (LV fractional shortening, LVFS), larger LV end-systolic diameter (LVESD), and thicker interventricular septum diameter (IVS) with similar LV end-diastolic diameter (LVEDD) than control rats. The alirocumab-treated HF rats had higher LVFS and smaller LVESD with similar IVS and LVEDD compared to HF rats (Figure 5B).

**Figure 6:**
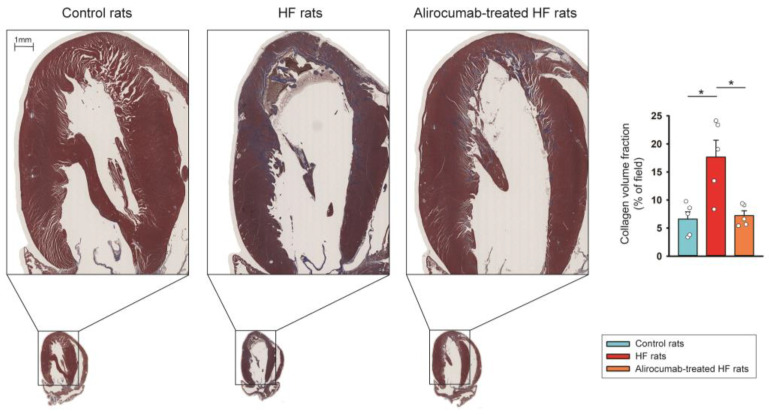
Anti-fibrotic effect of proprotein convertase subtilisin/kexin type 9 (PCSK9) inhibitor (alirocumab) on rats with isoproterenol-induced heart failure (HF). Photographs, individual data points, and averaged data reveal cardiac fibrosis (stained with blue color) studied using Masson’s trichrome staining in the left ventricular (LV) tissues from different groups. Control rats (*n* = 5 rats) and alirocumab-treated HF rats (*n* = 5 rats) exhibited less severe LV fibrosis than HF rats (*n* = 5 rats). The fibrosis levels of LV tissues were expressed as the collagen volume fraction, that is, the ratio of the LV total collagen surface area stained blue to the LV total surface area. * *p* < 0.05.

Masson’s trichrome staining revealed that HF rats had higher LV fibrosis than control rats, and alirocumab attenuated LV fibrosis significantly in HF rats (Figure 6). There is no significant difference in the serum level of total cholesterol and triglyceride between each group (Table 1).

## 3. Discussion

Genetic disruption and pharmacological inhibition of PCSK9 have been proven to decrease pulmonary fibrosis, liver fibrosis, and renal fibrosis [20,21,22], but the underlying mechanisms of the pro-fibrogenic effect have not been fully elucidated. To our knowledge, this is the first study reporting that PCSK9 enhanced NLRP3 inflammasome signaling through directly binding with TLR4 in human cardiac fibroblasts, thereby activating their pro-fibrotic cellular activities. Inhibition of NLRP3 by immunomodulator can attenuate the collagen production capability of cardiac fibroblasts [23]. Additionally, a previous study revealed MCC950, an NLRP3 inhibitor, decreased collagen production, myofibroblast differentiation of cardiac fibroblasts, and attenuated cardiac fibrosis in mice with HF [24,25]. In this study, we also found that MCC950 attenuated PCSK9-increased pro-fibrotic cellular activities, suggesting that PCSK9 activates fibroblast activities through NLRP3 signaling.

NLRP3 inflammasome activation arises from TLR4 signaling, which triggers the transcription of NLRP3 and pro-IL-1β. Subsequently, DAMPs activate the NLRP3 inflammasome, thereby facilitating the conversion of pro-IL-1β into IL-1β, amplifying downstream inflammatory signals [26]. In this study, we found that PCSK9 enhanced expressions of IL-1β in cardiac fibroblasts, which was attenuated by MCC950 co-treatment. Genetically knocking down PCSK9 can decrease IL-1β expression of ischemic myocardium [27]. Human recombinant PCSK9 can upregulate the expression of IL-1β in cardiomyocytes [28], suggesting that PCSK9 is an activator of the NLRP3 signaling pathway.

In mice with pressure-overload remodeling, cardiac NLRP3 inflammasome activation enhances greater myocardial fibrosis by the upregulation of TGF-β1 triggered by IL-1β [29]. IL-1β has been found to activate collagen production or myofibroblast differentiation capabilities of fibroblasts [30]. We found that PCSK9-treated cardiac fibroblasts had greater α-SMA protein expression, which was also reduced by MCC950, suggesting that PCSK9 increases the myofibroblast differentiation amount through the upregulation of NLRP3 signaling, thereby activating pro-fibrotic cellular activities.

**Figure 7:**
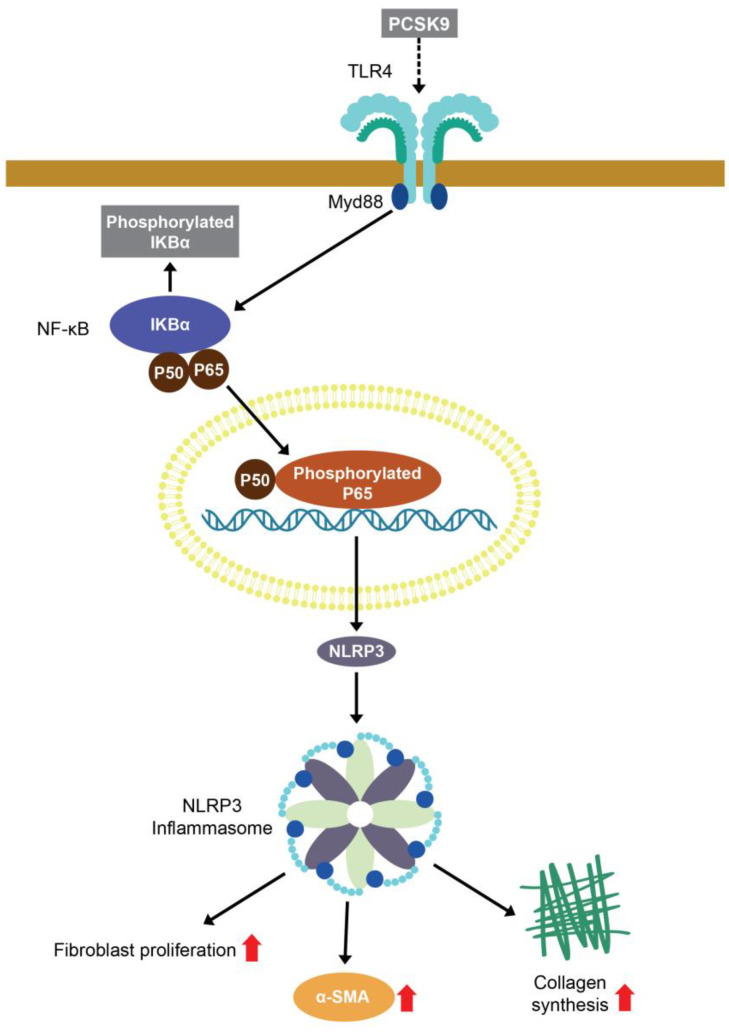
The proposed molecular mechanism underlying the pro-fibrotic effects of proprotein convertase subtilisin/kexin type 9 (PCSK9) on cardiac fibroblasts. PCSK9 activates Toll-like receptor 4 (TLR4)/Myd88/NF-κB signaling, thereby upregulating nucleotide-binding domain (NOD)-like receptor protein 3 (NLRP3) signaling and the profibrotic activities of cardiac fibroblasts.

TLR4 expression is higher in patients with severe HF than in those with stable HF [31,32]. TLR4 knockout HF mice exhibit less cardiac fibrosis compared to wild-type HF mice [33]. These findings suggest a crucial role of TLR4 in the pathogenesis of HF [32,34,35]. PCSK9 increases the pro-inflammatory tissue factor secretion capability of monocytes through TLR4 signaling [36,37], and PCSK9 overexpression can upregulate TLR4 protein expression on macrophages [18]. However, we found that PCSK9-treated fibroblasts exhibited similar levels of TLR4 protein expression compared to control cells, suggesting that PCSK9 does not activate TLR4 signaling through the modulation of TLR4 protein expression. Macrophages treated with PCSK9 exhibited internalization of the LDL receptor and TLR4 from the cell surface, thereby activating the TLR4 signaling pathway. These findings suggest that TLR4 activation may arise from direct interaction with PCSK9 on the cell surface or internalized PCSK9-LDLR complex [38]. Another study has shown a distinct structural similarity between the C-terminus cysteine-rich domain (CRD) of PCSK9 and the resistin homotrimer, a pro-inflammatory factor associated with obesity [39]. Resistin can directly bind with TLR4 protein through the loops of the C-terminal part, thereby activating TLR4 signaling [40,41]. Following TLR activation, the adapter protein MyD88 is recruited and triggers a cascade of signaling events that eventually lead to the phosphorylation of NF-κB-IκBα and NF-κB-p65 translocation to the nucleus, thereby promoting the expression of NLRP3 and pro-IL-1 [42,43]. The MyD88-independent TLR activation, also known as the TRIF-dependent pathway, is initiated through the TRIF protein and activates the NF-κB signaling [42]. This study found that PCSK9 had no significant effect on TRIF protein expression. PCSK9 upregulated the expression of Myd88, the ratio of phosphorylated IκBα, p65, NLRP3, and IL-1 which were downregulated by the TLR inhibitor. As summarized in Figure 7, PCSK9 activated TLR4/Myd88/NF-κB signaling, thereby upregulating the NLRP3 signaling pathway and pro-fibrotic cellular activities.

A previous HF trial revealed that circulating PCSK9 is elevated in patients with HF and is positively correlated with the prognosis of HF [9]. The serum level of PCSK9 is positively correlated with liver fibrosis [44]. Alirocumab has been proven to decrease pulmonary fibrosis in bleomycin-treated mice [20]. In the present study, we found that the serum level of PCSK9 was elevated 14 days after HF induction and that alirocumab reduced ventricular fibrosis in isoproterenol-treated rats. A pilot study revealed that a higher plasma level of PCSK9 was associated with an impairment of systolic function in patients post-MI [45]. Exogeneous PCSK9 decreases mitochondria biogenesis, thereby inducing apoptosis of cardiomyocytes [46,47,48]. Inhibition of PCSK9 improves the cell-shortening capability of cardiomyocytes [49]. Moreover, isolated perfused hearts from PCSK9 knockout mice exhibited better cardiac function compared with those from wild-type mice post-ischemic reperfusion injury [49]. In the present study, we found that alirocumab increased the LV systolic function of HF. In this study, we treated cardiac fibroblasts with 100 ng/mL, which is similar to that from the minimal plasma level in elders with comorbidity [50]. PCSK9 can be secreted by human fibroblasts [51]. However, we did not measure the level of PCSK9 in condition medium of human cardiac fibroblasts. Since the plasma levels of PCSK9 between healthy and HF rats were around 100 ng/mL, we treated human cardiac fibroblasts with 100 ng/mL of PCSK9 to mimic the different PCSK9 plasma levels between healthy and HF rats. Thus, the pro-fibrogenic effect of PCSK9 in human cardiac fibroblasts found in the current study is thought to be clinically relevant, and PCSK9 may be a potential therapeutic target for HF.

There are some limitations in this study. First, we found that both the TLR4 inhibitor (TAK242) and the NLRP3 inhibitor (MCC950) attenuate PCSK9-activated pro-fibrotic cellular activities. Although TAK242 and MCC950 have demonstrated minimal off-target effects and have been extensively validated in vitro [52,53,54,55], the possibility of off-target interactions cannot be entirely excluded. Future studies should consider genetic knockdown approaches to further validate the involvement of these pathways. Second, we found that PCSK9 can activate TLR4/Myd88 signaling in cardiac fibroblasts. However, this study did not conduct a reporter or co-immunoprecipitation assay. Hence, it is not clear whether the activation of TLR4 signaling is mediated through PCSK9-TLR4 binding directly or another mediator between PCSK9 and TLR4. Third, we studied collagen and α-SMA protein expression 48 h after PCSK9 treatment, but evaluated the proliferation rate 24 h after treatment. We found that compared to control cells, PCSK9-treated fibroblasts exhibited higher collagen and α-SMA expression 48 h post-treatment and a higher proliferation rate 24 h post-treatment. A previous study revealed that activated myofibroblasts with highly expressed α-SMA produces collagen mostly from 24 to 48 h [56]. Our study aims to understand the biological effect of PCSK9 on fibrosis and collagen production. We did not perform protein analysis 24 h after treatment in our cellular models. Pathways participating in the proliferation process (including Ca^2+^, MAPK, PI3K/Akt/mTOR) are not completely the same as the pathways involved in PCSK9-induced collagen production [57]. It is not clear whether protein expression is already different 24 h post-treatment. Fourth, previous studies revealed that in rats post-ischemic reperfusion, myocardial PCSK9 attained its peak at 2 weeks post-reperfusion following a significant decrease at 3 or 4 weeks [58,59]. In rats with surgically induced MI, serum PCSK9 levels reach their peak at 48 h and subsequently decrease at 96 h [60]. In our study, we found that the levels of PCSK9 were significantly elevated 14 days after HF induction. However, another study revealed that no matter whether in rats or humans, increased plasma PCSK9 levels were associated with LV systolic dysfunction [61]. We found that the LV systolic function of HF rats on the 28th day is still lower than that of healthy rats, but the serum levels of PCSK9 are not evaluated in the studied animals. Fifth, while alirocumab was shown to improve cardiac fibrosis in rats with isoproterenol-induced HF, a well-established HF model [62], the anti-fibrotic effects of alirocumab were not assessed in rats with HF following occlusive MI. A prior study has shown that another PCSK9 inhibitor, evolocumab, attenuated myocardial fibrosis in rats with ischemia reperfusion injury and HF [58], supporting the findings of our study.

## 4. Materials and Methods

### 4.1. Cell Cultures

Human cardiac fibroblasts, sourced from Lonza Research Laboratory (Walkersville, MD, USA) were cultured as monolayers in uncoated dishes, utilizing the FGM™-3 Cardiac Fibroblast Growth Medium-3 BulletKit (Lonza Research Laboratory). The culture conditions were maintained at a temperature of 37 °C in an environment with 5% CO_2_ concentration. To mitigate potential disparities in cellular function, only cells from passages 4 to 6 were used in the experiments. Our cell study protocols adhered to the Declaration of Helsinki and received approval from the local institute ethics committee (TMU-JIRB No. N202404144).

### 4.2. Cell Migration Assay

As shown in Supplementary Figure S1, the migration capability of cardiac fibroblasts was assessed using a wound-healing assay. Cells were first cultured in 6-well plates and maintained in serum-free medium for 72 h. After 24 h in serum-free conditions, cells were treated with PCSK9 (50 or 100 ng/mL; R & D Systems, Minneapolis, MN, USA) for 48 h. Six hours before the end of treatment, a gap was created by scraping the cells with a P200 pipette tip to initiate migration analysis. The net migration area, determined using Image J 1.45s software (National Institute of Health, Bethesda, MD, USA), represented the difference between the initial and six-hour post-scratch gap areas.

### 4.3. Cell Proliferation Assay

Cardiac fibroblast proliferation was assessed through an MTS assay (Promega, Madison, WI, USA) as previously described [63]. In brief, cardiac fibroblasts were seeded at a density of 3000 cells/well onto a 96-well culture dish. Once 50% confluence was attained, the cells were treated with PCSK9 (50 or 100 ng/mL), the NLRP3 inhibitor MCC950 (100 μmol/L, MedChem Express, Monmouth Junction, NJ, USA), or the TLR4 inhibitor TAK242 (10 μmol/L, Cayman Chemical, Ann Arbor, MI, USA) in culture medium for 24 h. Cell growth was analyzed using the MTS reagent four hours before spectrophotometric measurement.

### 4.4. Western Blotting

Western blotting was done as described previously [64]. Cardiac fibroblasts treated with or without PCSK9 (50 or 100 ng/mL), and cells treated with MCC950 (100 μmol/L) or TAK242 (10 μmol/L) for 48 h were lysed using radioimmunoprecipitation assay buffer containing 150 mmol/L NaCl, Nonidet P-40, 50 mmol/L Tris at pH 7.4, 0.5% sodium deoxycholate, 0.1% sodium dodecyl sulfate (SDS), and protease inhibitor cocktails supplied by Sigma-Aldrich (St. Louis, MO, USA). We separated the proteins using 10% SDS-polyacrylamide gel electrophoresis and subsequently transferred them to an equilibrated polyvinylidene difluoride membrane (Amersham Biosciences, Buckinghamshire, UK). Fractionated protein was probed using primary antibodies against α-SMA (1:5000, monoclonal, clone number: 1A4, Abcam, Cambridge, UK), pro-collagen type IA1 (1:500, monoclonal, clone number: 3G3, Santa-Cruz Biotechnology, Santa Cruz, CA, USA), NLRP3 (1:1000, polyclonal, cell signaling, Beverly, MA, USA), IL-1β (1:2000, polyclonal, Abcam), TLR4 (1:2000, monoclonal, clone number: 9q33.1, Santa-Cruz Biotechnology), Myd88 (1:1000, monoclonal, clone number: D80F5, cell signaling), TRIF (1:1000, polyclonal, proteintech, Wuhan, China), phosphorylated p65 (1:1000, monoclonal, clone number: 93H1, cell signaling), total p65 (1:1000, monoclonal, clone number: D14E12, cell signaling), phosphorylated IκBα (1:1000, polyclonal, Bioss Antibodies, Boston, MA, USA), and total IκBα (1:1000, polyclonal, Santa-Cruz Biotechnology) followed by incubation with secondary antibodies conjugated with horseradish peroxidase. Bound antibodies were identified using an enhanced chemiluminescence system (Millipore, Darmstadt, Germany) and analyzed with AlphaEaseFC version 4.0 software (Alpha Innotech, San Leandro, CA, USA). Glyceraldehyde 3-phosphate dehydrogenase (GAPDH) protein (Sigma-Aldrich), as a loading control, confirmed equal protein loading and was then normalized to the value of control cells.

### 4.5. Effects of PCSK9 Inhibitor on Cardiac Function and Fibrosis in HF Animals

We did HF induction as described previously [65]. Male Wistar rats (10 weeks old, weighing 300−350 g) were subcutaneously administered a single subcutaneous high dose of isoproterenol (100 mg/kg). Two days after injection, the LVFS of these rats was evaluated using echocardiography. We included the rats with LVFS < 45% in the HF group [66]. HF rats were then randomly treated using intra-peritoneal Alirocumab (10 mg/kg/week for 28 days, Praluent, Sanofi, Paris, France) or vehicle injection. Following the 28-day treatment, we euthanized both the treated rats and their age-matched healthy male controls using an overdose of 5% isoflurane (in oxygen) for histological examination. Our animal study protocols adhered to the ARRIVE guidelines and Guide for the Care and Use of Laboratory Animals by the US National Institutes of Health (NIH Publication No. 85-23, revised 2011) and received approval from the Taipei Medical University animal ethics committee (LAC2024-0100).

Rats were sedated with 2% isoflurane (in oxygen) and scanned using a Vivid i ultrasound cardiovascular system echo scanner (GE Healthcare, Haifa, Israel) and a 10S phased array pediatric transducer before euthanasia. The transmission frequency was 10 MHz; the depth 2.5 cm; and the frame rate 225 frames/s. Rats were placed in a supine position. LVEDD, LVESD, and LV IVS were acquired under parasternal mid-papillary short-axis view using M-mode imaging (Supplementary Table S1). The LVFS (%) was measured as (LVEDD-LVESD)/LVEDD × 100.

For Serum PCSK9 analysis, blood serum was collected before and 14 days after isoproterenol injection and assayed for PCSK9 using a PCSK9 fluorometric assay kit (Cayman Chemical Co., Ann Arbor, MI, USA) according to the manufacturer’s instructions.

### 4.6. Serum Lipid Profile Analysis

For serum lipid profile analysis, blood serum was collected before euthanasia. Serum levels of total cholesterol and triglyceride were analyzed using an IDEXX Catalyst One instrument (IDEXX Laboratories, Inc., Westbrook, ME, USA).

### 4.7. Cardiac Fibrosis Analysis in HF Rats

Cardiac fibrosis analysis was done per a previously described method with modification [65]. Briefly, LV tissues were fixed in 4% formaldehyde, paraffin-embedded, and stained using Masson’s trichrome. Bright-field images were captured for these tissues. LV fibrosis was quantified by calculating the collagen volume fraction, which is the ratio of total collagen surface area to total LV surface area. Collagen deposition across the entire sectioned LV tissues was evaluated blindly using HistoQuest Analysis Software (version 4.0, TissueGnostics, Vienna, Austria).

### 4.8. Statistical Analysis

All quantitative data were expressed as mean ± standard error of the mean. Statistical analysis was conducted exclusively with a minimum group size of *n* = 5. The declared group size corresponds to the number of independent values (biological replicates, not technical replicates), which were used for the statistical analysis in this study. Animals were randomized to ensure equal group sizes and allocated to either healthy control, HF with vehicle treatment, or alirocumab for studies on cardiac structure and systolic function. Analysis was performed by the observer in a blinded manner. Statistical analyses began with the use of the Shapiro–Wilk test to evaluate normality. A paired *t*-test for normal distribution, a one-way repeated-measures ANOVA with a post hoc Fisher’s least significant difference (LSD) test for normal distribution only if F in ANOVA achieved *p* < 0.05, a Friedman test with a post hoc Wilcoxon sign rank test, and a Kruskal–Wallis test with a post hoc Mann−Whitney rank-sum test for non-normal distribution were used to compare cells and rats under different conditions. A *p* < 0.05 was considered statistically significant.

## 5. Conclusions

In conclusion, PCSK9 upregulates NLRP3 signaling and the profibrotic activities of cardiac fibroblasts through the activation of TLR4/Myd88/NF-κB signaling.

## Figures and Tables

**Table 1:** Serum levels of total cholesterol and triglyceride from each group.

Serum	HF Groups (*n* = 6)	HF Groups with Alirocumab (*n* = 6)	Healthy Control Groups (*n* = 6)
Total cholesterol (mg/L)	470.0 ± 55.9	471.7 ± 65.2	463.3 ± 37.0
entry 2	840.0 ± 135.5	846.7 ± 102.0	900.0 ± 82.0

HF: heart failure.

## Data Availability

The data analyzed during the current study are available from the corresponding authors upon reasonable request.

## Supplementary Materials

The following supporting information can be downloaded at: https://www.mdpi.com/article/10.3390/ijms26051921/s1.
