# Supplementary material for: PCSK9 Enhances Cardiac Fibrogenesis via the Activation of Toll-like Receptor and NLRP3 Inflammasome Signaling
Source: Int J Mol Sci. 2025 Feb 23;26(5):1921. doi: 10.3390/ijms26051921 (PMC11900342; doi:10.3390/ijms26051921)
Supplement: Supplementary file 1 [file ijms-26-01921-s001.zip › ijms-3437815-supplementary.pdf]

Type of the Paper (Article)

PCSK9 Enhances Cardiac Fibrogenesis via the Activation of Toll-like Receptor and NLRP3 Inflammasome Signaling

Cheng-Chih Chung [1,2,3], Yu-Hsun Kao [4,5], Yao-Chang Chen [6], Yung-Kuo Lin [1,2,3], Satoshi Higa [7], Kai-Cheng Hsu [8,9], and Yi-Jen Chen [1,2,3,4,*]

[1] Division of Cardiology, Department of Internal Medicine, School of Medicine, College of Medicine, Taipei Medical University, Taipei, Taiwan; michaelchung110@gmail.com (C.C.C); yklin213@yahoo.com.tw (Y.K.L); yjchen@tmu.edu.tw (Y.J.C)
[2] Division of Cardiovascular Medicine, Department of Internal Medicine, Wan Fang Hospital, Taipei Medical University, Taipei, Taiwan
[3] Taipei Heart Institute, Taipei Medical University, Taipei, Taiwan
[4] Graduate Institute of Clinical Medicine, College of Medicine, Taipei Medical University, Taipei, Taiwan; yuhsunkao@gmail.com (Y.H.K)
[5] Department of Medical Education and Research, Wan Fang Hospital, Taipei Medical University, Taipei, Taiwan
[6] Department of Biomedical Engineering, National Defense Medical Center, Taipei, Taiwan; yao-chang.chen@gmail.com (Y.C.C)
[7] Cardiac Electrophysiology and Pacing Laboratory, Division of Cardiovascular Medicine, Makiminato Central Hospital, Okinawa, Japan; sa_higa@yahoo.co.jp (S.H)
[8] Graduate Institute of Cancer Biology and Drug Discovery, College of Medical Science and Technology, Taipei Medical University, Taipei, Taiwan; piki@tmu.edu.tw (K.C.H)
[9] Ph.D. Program for Cancer Molecular Biology and Drug Discovery, College of Medical Science and Technology, Taipei Medical University, Taipei, Taiwan
* Yi-Jen Chen, MD, PhD (e-mail: *yjchen@tmu.edu.tw*) or Kai-Cheng Hsu, PhD (e-mail: piki@tmu.edu.tw) Graduate Institute of Clinical Medicine, College of Medicine, Taipei Medical University, No. 250, Wu-Hsing Street, Taipei 11031, Taiwan. Tel:+886 02-2736-1661#3028

Figure S1. Schematic summarizing the treatment protocol of the migration analysis for cardiac fibroblasts.

Table S1. Raw data of rat cardiac structure recorded with echocardiography

Number of rats	IVS (mm)	LVEDD (mm)	LVESD (mm)
Healthy Rat No.1	1.8	7.0	2.9
Healthy Rat No.2	1.3	8.6	3.8
Healthy Rat No.3	1.6	7.6	3.5
Healthy Rat No.4	2.1	7.3	3.2
Healthy Rat No.5	1.9	6.8	2.6
Healthy Rat No.6	1.8	7.6	3.3
HF Rat No.1	2.3	6.9	4.0
HF Rat No.2	2.8	7.7	5.5
HF Rat No.3	2.3	8.4	6.1
HF Rat No.4	2.1	9.5	5.7
HF Rat No.5	2.2	7.6	4.8
HF Rat No.6	2.5	8.0	5.0
HF Rat with Alirocumab No.1	1.6	7.7	3.2
HF Rat with Alirocumab No.2	1.9	8.5	4.6
HF Rat with Alirocumab No.3	3.3	7.0	3.3
HF Rat with Alirocumab No.4	2.0	8.8	4.6
HF Rat with Alirocumab No.5	2.2	8.0	4.5
HF Rat with Alirocumab No.6	2.5	6.7	2.5

IVS: interventricular septum diameter; *LVEDD*: left ventricular end diastolic diameter; *LVESD*: left ventricular end systolic diameter; *HF*: heart failure
